# Online Information About Periviable Birth: Quality Assessment

**DOI:** 10.2196/12524

**Published:** 2019-06-07

**Authors:** Adriane F Haragan, Carly A Zuwiala, Katherine P Himes

**Affiliations:** 1 Department of Obstetrics and Gynecology Kalispell Regional Healthcare Kalispell, MT United States; 2 Department of Obstetrics and Gynecology Magee-Womens Hospital University of Pittsburgh Pittsburgh, PA United States; 3 Department of Obstetrics, Gynecology and Reproductive Sciences Magee-Womens Research Institute University of Pittsburgh Pittsburgh, PA United States

**Keywords:** periviable birth, patient education, patient counseling, Internet resources

## Abstract

**Background:**

Over 20,000 parents in the United States face the challenge of participating in decisions about whether to use life support for their infants born on the cusp of viability every year. Clinicians must help families grasp complex medical information about their baby’s immediate prognosis as well as the risk for significant long-term morbidity. Patients faced with this decision want supplemental information and frequently seek medical information on the Internet. Empirical evidence about the quality of websites is lacking.

**Objective:**

We sought to evaluate the quality of online information available about periviable birth and treatment options for infants born at the cusp of viability.

**Methods:**

We read a counseling script to 20 pregnant participants that included information typically provided by perinatal and neonatal providers when periviable birth is imminent. The women were then asked to list terms they would use to search the Internet if they wanted additional information. Using these search terms, two reviewers evaluated the content of websites obtained via a Google search. We used two metrics to assess the quality of websites. The first was the DISCERN instrument, a validated questionnaire designed to assess the quality of patient-targeted health information for treatment choices. The second metric was the Essential Content Tool (ECT), a tool designed to address key components of counseling around periviable birth as outlined by professional organizations. DISCERN scores were classified as *low quality* if scores were 2, *fair quality* if scores were 3, and *high quality* if scores were 4 or higher. Scores of 6 or higher on the ECT were considered high quality. Interreviewer agreement was assessed by calculated kappa statistic.

**Results:**

A total of 97 websites were reviewed. Over half (57/97, 59%) were for-profit sites, news stories, or personal blogs; 28% (27/97) were government or medical sites; and 13% (13/97) were nonprofit or advocacy sites. The majority of sites scored poorly in DISCERN questions designed to assess the reliability of information presented as well as data regarding treatment choices. Only 7% (7/97) of the websites were *high quality* as defined by the DISCERN tool. The majority of sites did not address the essential content defined by the ECT. Importantly, only 18% of websites (17/97) indicated that there are often a number of reasonable approaches to newborn care when faced with periviable birth. Agreement was strong, with kappa ranging from .72 to .91.

**Conclusions:**

Most information about periviable birth found on the Internet using common search strategies is of low quality. News stories highlighting positive outcomes are disproportionately represented. Few websites discuss comfort care or how treatment decisions impact quality of life.

## Introduction

While the rate of survival with or without neurodevelopmental impairment for infants born between 22 and 25 weeks has improved over time, there is considerable uncertainty regarding short- and long-term prognosis for infants born during the periviable period [1-8]. Periviable birth, defined as delivery occurring from 20 0/7 weeks to 25 6/7 weeks, complicates roughly 20,000 deliveries annually in the United States. Survival with neonatal interventions ranges from roughly 10% at 22 weeks gestation to over 60% at 25 completed weeks [1]. The risk of long-term neurodevelopmental impairment remains high for all periviable infants who survive [1-5]. Women and families must navigate this uncertainty to make time-sensitive and value-laden decisions regarding obstetrical interventions for fetal benefit and neonatal care when periviable birth is imminent. Women facing periviable birth have expressed a desire for supplemental information after provider counseling [9-13].

Increasingly, patients are turning to the Internet for supplemental health information. Survey estimates suggest that over half of people in the United States search the Internet for health information [14]. While data specific to periviable birth are limited, studies of other preference-sensitive decisions suggest that both patients and providers support provision of supplemental information that presents the pros and cons of all reasonable treatment options. Health care providers remain skeptical about the value of the Internet as a source of unbiased supplemental information [11,15].

The usefulness of the Internet as a source of supplemental health information depends on the quality of information easily available to patients. There are now a number of validated assessment checklists—the DISCERN tool, Journal of American Medical Association Benchmarks, and Health On the Net Foundation Principles—to determine the quality of online information [16-18]. The aim of this study was to evaluate data regarding the quality of online information about periviable birth.

## Methods

### Defining Internet Search Terms

We recruited pregnant women at gestation of 37 weeks or more from the Magee-Womens Hospital, Pittsburgh, PA, outpatient obstetrical clinics to participate in this study during June 2016. Women were excluded if they had experienced a prior preterm birth or had experienced threatened preterm labor during their current pregnancy. We approached all women meeting inclusion criteria. Following informed consent, women were read a script describing a hypothetical situation in which they were faced with a preterm birth at 23-weeks’ gestation. The script included information on short- and long-term outcomes, risks and benefits of obstetrical interventions for neonatal benefit, as well as care options for the neonate after delivery. The script was developed by input from members of the division of Maternal Fetal Medicine and Newborn Medicine at the University of Pittsburgh Medical Center, Pittsburgh, PA. Participants were then given a written survey asking them to identify the search engine they used most frequently and how often they searched for health information online. They were then asked to list four search terms they would use to seek out additional information about periviable birth. They were also asked whether they would prefer to learn about (1) statistics regarding survival and outcomes for periviable neonates, (2) narrative descriptions about women who delivered in the periviable period and the outcomes for their neonates, or (3) both. Women were recruited until we reached saturation in themes of search terms. We did not recruit partners of pregnant women as they were not readily present at the clinical venues where we recruited participants.

### Website Identification

Using the four most common search terms provided, two reviewers (AFH and CAZ) independently evaluated the informational content of websites found via a Google search. Additional websites listed by participants were WebMD and Wikipedia. Sites were excluded if they were non-English or if registration was required for access. Eligible websites were then independently reviewed on the same date. The first three pages of websites using the top four search terms were included in the evaluation, as marketing data demonstrate that only 1.6% of users click on links from the third page of results or beyond [19].

### Analysis of Website Quality

Websites were classified into six main categories: (1) government, (2) scientific resources, (3) nonprofit and advocacy organizations, (4) news and media reports, (5) for-profit organizations, and (6) personal commentary (eg, personal blogs). Two metrics were used to evaluate the quality and content of the websites. The first was the DISCERN instrument, a validated questionnaire designed to assess the quality of patient-targeted health information for treatment choices [20]. We elected to use this tool because it is tailored to evaluate how well websites prepare patients and providers to engage in a shared decision-making process, the approach recommended to help families make decisions about neonatal care after periviable birth. Questions included in the DISCERN instrument fall into three main sections. Section 1 (Questions 1-8) addresses the reliability of the site and whether it can be trusted as a source of information about treatment choices. These questions address the sources of information used to compile the site, the website’s ability to provide accurate and impartial information, and areas of uncertainty regarding treatment choices. Section 2 (Questions 9-15) focuses on specific details of the information about treatment choices. These questions examine the risks and benefits of each treatment choice and how well the site supports shared decision making. Section 3 (Question 16) is a subjective assessment by the reviewer of the overall quality of the website. Each question on the DISCERN tool is scored on a scale of 1 (low quality/not addressed) to 5 (high quality/fully addressed). We considered scores of 4-5 to be *high quality*, 3 to be *fair quality*, and 1-2 to be *poor quality* sources of information.

The DISCERN tool can be used for any health-related content area and, thus, is not specific to periviable birth. Therefore, a second metric was developed—the Essential Content Tool (ECT)—to address whether websites covered information defined as critical for decision making around periviable birth. This tool was developed in two phases. First, we extracted 21 key components of periviable counseling as outlined by both the American College of Obstetrics and Gynecology and the American Academy of Pediatrics statements about periviable birth [21,22]. Content validity was evaluated by querying four physician members of the divisions of Maternal Fetal Medicine and Newborn Medicine to identify the minimum number of constructs necessary to provide families with essential information about periviable birth decision making. This process identified 10 essential topics pertinent to periviable birth. These included information about short- and long-term neonatal morbidity and mortality at different gestational ages as well as obstetrical interventions for neonatal benefit and options for neonatal care. We also assessed whether information was presented in a preference-sensitive manner: there is no right or wrong decision. All 10 domains are outlined in Multimedia Appendix 1. Thus, the ECT contains a total of 10 content questions that were scored dichotomously (present or not present). A website was considered of *good quality* if it addressed at least six of the content domains and *high quality* if it addressed at least eight of the content domains.

### Statistical Analysis

Descriptive statistics were calculated to characterize participants and summarize results of the DISCERN tool and the ECT. Interreviewer agreement was assessed by a calculated kappa statistic and descriptive statistics were performed. This study was approved by the University of Pittsburgh’s Institutional Review Board.

## Results

### Patient Population and Search Terms

A total of 20 women were recruited for the first stage of the study. The median age of our patient population at the time of enrollment was 28 years (interquartile range [IQR] 25-30), 35% (7/20) had Medicaid insurance, 65% (13/20) had private insurance, and 50% (10/20) of the women were black. A total of 90% (18/20) of women had graduated high school. The median gestational age was 38.0 weeks (IQR 37.6-38.6) and 40% (8/20) were nulliparous. Of those surveyed, 85% (17/20) chose Google as their preferred search engine and 75% (15/20) reported they used the Internet to search for health information *often* or *all the time*.

Patients were asked to provide four search terms they would use to search for supplemental information online regarding periviable birth. Overall, participants recorded a total of 54 search terms. The four most common search terms elicited were (1) preterm birth, (2) birth at 23 weeks, (3) long-term effects of preterm birth, and (4) chances of survival at 23 weeks. These four responses or minor variations of these responses (ie, long-term complications of preterm birth) accounted for 83% (45/54) of total search terms provided by participants. Other search terms recorded by participants included the following: recurrence risk of preterm birth, prevention of preterm birth, resources available for people with preterm infants, and care decisions made by families facing periviable birth. Patients overwhelmingly desired both statistical information about survival and prognosis as well as patient narratives about their experiences with periviable birth, with 90% of women indicating they wanted both types of information.

### Website Characteristics

After exclusion criteria were applied, 97 unique websites were reviewed out of a total of 120 possible websites (80.8%). Of those queried, 59% (57/97) were for-profit sites, news stories, or personal blogs; 28% (27/97) were government, hospital or research institution, or medical journal sites; and 13% (13/97) were nonprofit or advocacy sites. Most websites were from the United States (75/97, 77%), followed by the United Kingdom (17/97, 18%), Australia (3/97, 3%), and New Zealand (2/97, 2%).

### Quality of Website Content: The DISCERN Tool

The DISCERN tool evaluates the reliability of website information as well as the quality of information about treatment choices. The reviewers defined “treatment” as neonatal interventions with the goal of sustaining the life of the neonate. Therefore, when the DISCERN tool asks if the website “addresses what would happen if no treatment was used or if there are alternatives to treatment,” we interpreted this as the website referring to the option of comfort care and subsequent neonatal death.

**Figure 1:**
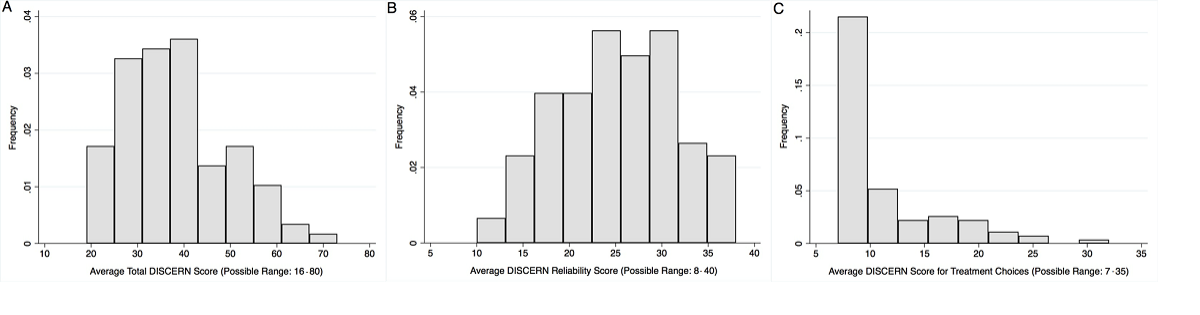
Distribution of DISCERN scores overall (A), with regard to reliability of information (B) and information on treatment choices (C).

Overall, websites scored poorly. A website could score a total of 80 points on 16 questions, 40 points on questions addressing the reliability of information (Questions 1-8), and 35 points on the questions addressing treatment choices (Questions 9-15). High scores indicate *high quality*. The distribution of scores is shown in Figure 1. While website quality was poor overall (see Figure 1A) with a median overall score of 36 (IQR 30-44), websites fell markedly short at discussing treatment options (see Figure 1C). The median score for the questions addressing treatment choices was 8 (IQR 7-13).

As indicated above (see Figure 1C), information on treatment choices was poor and this was particularly notable for questions addressing support for shared decision making. Only 10% (10/97) of the websites scored highly (4 or 5) on the question of whether it was clear there was more than one reasonable treatment choice or medical approach. A total of 68% of websites (66/97) scored poorly (<2) in acknowledging uncertainty around the best treatment options. Importantly, 16% (16/97) had evidence of strong bias indicating a completely unbalanced view of options available to patients experiencing a periviable birth. The bias was uniformly in favor of presenting the option of a trial of resuscitation and not presenting comfort care as an option.

### Website Content Using the Essential Content Tool

The ECT provides a more granular assessment of the quality of information as it pertains to periviable birth. Consistent with the findings using the DISCERN tool, most websites lacked the essential content necessary for periviable decision making as defined by the ECT. Only 38 of the 97 sites (39%) reviewed morbidity and mortality statistics by gestational age, and these were often not reflective of the current literature [1]. Roughly a quarter (27/97, 28%) reviewed obstetrical interventions for neonatal benefit, such as administration of betamethasone or magnesium for neuroprotection. Interestingly, the content area most commonly addressed by the queried websites (59/97, 61%) was the potential long-term outcomes for periviable neonates (eg, cognitive impairment, cerebral palsy, deafness, blindness, feeding difficulties, and oxygen requirement). Consistent with our findings from the DISCERN tool, only 18% (17/97) of websites presented a full range of care options for periviable infants, including comfort care. Few websites (10/97, 10%) characterized decision making around periviable birth as preference-sensitive, indicating that there is often no clear right or wrong choice in deciding to pursue life-sustaining interventions versus comfort care for the neonate. The complete distribution of scores for ECT are included in Multimedia Appendix 1.

### Overall Website Quality

The overall quality of website content was measured using the last question on the DISCERN tool and by the presence of six out of 10 essential domains as defined by the ECT. Only 7% (7/97) of the websites were thought to represent high-quality websites by both reviewers using the last question on the DISCERN tool, and 2% (2/97) were considered high quality according to the ECT. A total of 20% (19/97) of the websites were of good quality according to the ECT. Notably, over half (5/9, 56%) of the high-quality websites were found by our reviewers on page 2 or 3 of the search results. Additionally, 74% (14/19) of good-quality websites were from academic sources, governmental sources, or nonprofit organizations. Only 2 out of 19 (11%) of the good-quality websites according to the ECT were from a for-profit site. To illustrate high- and low-quality websites, we provided quotes demonstrative of the websites’ messages. We found that many websites, particularly news stories and personal blogs, focused on sensational stories that do not always represent the more likely outcomes (see Table 1).

Interreviewer agreement was substantial with kappa ranging from .72 to .90 for the ECT questions and from .36 to .71 for the individual DISCERN questions. The stronger agreement within the ECT can be explained by the binary nature of the questions, while the DISCERN questions are answered using a 1-5 scale. When using a weighted kappa statistic to account for the degree of disagreement within the DISCERN questions, agreement was strong, with kappa ranging from .66 to .91 for the individual DISCERN questions.

**Table 1:** Content from high- and low-quality websites.

Characteristics by DISCERN tool		Representative quotes
**High-quality website**		
	Address both survival and long-term disabilities	“For babies born at 23 or 24 weeks, the chance of survival if they receive intensive treatment is about 50:50. If the baby survives, they may have one or more of the problems described in this website...About 1 in 4 or 1 in 5 children who survive have very serious problems affecting their movement or learning or both that mean they will need lifelong help and support for everyday activities.” [23] “The success of improved survival in very premature infants has raised some serious ethical issues. It is now possible to save more, smaller, and earlier babies. The difficult question is whether this is always in the best interest of the baby...Decisions pertaining to these sensitive issues are influenced by a number of factors, not least by parental views.” [24]
	Address patients’ values	“Parents have difficult decisions to make at this time and your views and values are very important.” [23] “For some families, the worst thing that could happen is that their baby dies. For them, intensive treatment is the right choice as this gives the baby the best chance of surviving. For other families, the worst thing that could happen is that the baby has to go through intensive treatment and then survives with a serious disability. They worry about the effect on the child and on the rest of their family. For those families, comfort care may be the right choice. This option has the lowest chance of the baby surviving with a serious disability.” [25]
	Encourage shared decision making	“If you don’t know what would be best for your family and for your baby, you may find it helpful to talk to other members of your family. If there is time, you could speak to different medical specialists about your situation, eg, obstetricians and paediatricians.” [23] “Your doctors will talk with you about your situation and try to understand what is important for you and your family. There is no right or wrong answer.” [25]
**Low-quality website**		
	Sensational news stories	“With his chances of survival being between 15-30 percent, sweet Haiden has beat the odds...Within hours Emily gave birth to her 1.5-pound baby boy—14 hours away from the nearest hospital...Emily credits her boy’s strong lungs, a makeshift incubator and her cruise’s early arrival into a Puerto Rico dock. ‘The doctors really tell us that he’s a miracle baby,’ Emily stated. ‘It’s a miracle he’s here.’” “‘They didn’t think she was going to be alive, but I knew she was. Because I just knew it,’...She remembered the dream she had the night before going into labor. In it, she said God told her he would take care of her daughter, but she had to have ‘faith...’”
	Biased testimonials	“...studies show that depending on medical care, at 22 weeks—the age that Planned Parenthood is killing viable babies—preemies can survive with care. So, Trevor Frolek came into the world at 23 weeks. At the time of his birth, he weighed in at 1 pound, 6 ounces, and like many of the babies born alive in ‘botched’ abortions would do if given the chance, Trevor fought to stay alive. And stay alive he did. Trevor survived, and after spending the first year of his life in Fargo, North Dakota’s Essential Health neonatal intensive care unit, weighing a healthy 20 pounds, he went home.” “She was fully human, just smaller than we had ever seen before in our lives. Four months later, Ava Joy came home with us as a completely healthy baby with a minor case of reflux.”
	Statements with unclear sources of information	“A prematurity prevention program has been developed and implemented at the Pope Paul VI Institute for the last 25 years...This entire protocol cannot be properly discussed in a website such as this, however, it can be stated emphatically that the prematurity rate can be decreased with the use of this protocol...For the entire group, the comparison group had a preterm birth rate of 12.0 percent and the Pope Paul VI Institute group protocol only had a 7.0 percent prematurity rate and in that group, only 1.3 percent were at < to 33.9 weeks of gestation. This is three times less than the comparison group.”

## Discussion

### Principal Findings

The American College of Obstetricians and Gynecologists, the Society for Maternal Fetal Medicine, and the American Academy of Pediatrics all emphasize the need for shared decision making regarding interventions for infants born during the periviable period [21,22]. Given patients frequently access the Internet for supplemental health information when faced with medical decision making, we sought to provide data regarding the quality of online information as it pertains to periviable delivery [11,12]. Our work revealed that the overwhelming majority of websites do not address content considered essential for patient education about the difficult decisions surrounding periviable birth. Less than 20% of websites addressed comfort care as a treatment option in periviable neonates and, if mentioned, it was often a single sentence. Furthermore, only 10% of websites acknowledged that there is more than one reasonable approach to care—a frame that is critical to supporting shared decision making. Additionally, over 15% of websites were found to have strong bias in favor of neonatal resuscitation, potentially further eroding high preference-based decision making.

Roughly one in 10 websites were considered *high quality* by the DISCERN tool. The websites receiving the highest scores were nonprofit sites and government sites (eg, Wikipedia and the Australian Perinatal Practice Guidelines). Of concern, the highest-quality websites were often difficult to find within our search, often listed on the second or third page of the results. Prior research has shown that 91% of Internet users do not go beyond the first page of search results [15]. The websites that Google ranks on the first page of their search results for any given search term are those considered the most relevant using a complex algorithm, which is constantly updated and revised. Therefore, it can be difficult to move high-quality sites to the front of the search results without search engine optimization consultants [26].

Prior studies have investigated the quality of online information for other preference-sensitive decisions, including treatment for localized breast cancer and treatment options for prostate cancer [27,28]. Consistent with our results, these studies concluded that although many websites address these topics, very few provide essential information necessary to actively participate in decision making related to treatment options. These studies suggest that there is a potential for the Internet to provide valuable information for patients, but it is up to their health care providers to identify high-quality websites to guide their patients.

Our study has several important strengths. First, ours is the first investigation to specifically investigate the quality of online information as it pertains to periviable birth. Additionally, the search terms we used were defined by patients and thus likely reflect the usual search strategies employed by the general population. Furthermore, we used two metrics to evaluate the individual websites—both a validated tool to assess the quality of information for preference-sensitive decisions as well as a content tool that specifically assessed information related to periviable birth. This approach allowed for a multidimensional evaluation of the websites that addressed both content-specific as well as big-picture concepts that support high-quality shared decision making. Finally, interviewer agreement was found to be strong after our analysis.

Our study also has several important limitations. First, the Internet search terms used in our study were generated by pregnant women at term, rather than women facing imminent periviable birth. This approach was taken after extensive deliberation with the study team and review board. We weighed the risk of the added stress and anxiety that participating in this study would cause a woman facing periviable birth versus the minimal added benefit that would come from asking the intended population to generate the search terms for our study. At the conclusion of our discussions, we felt a hypothetical approach with term women would generate realistic and useful search terms and opted for this approach. Second, we attempted to recreate how the patients would search the Internet for additional information regarding treatment options and outcomes surrounding a periviable birth and thus only one search engine was used, which may limit the generalizability of our findings. This search engine, however, was overwhelmingly picked as the search engine of choice by our sample population and still provided a large amount of pilot data. Third, the review of the websites was completed on personal computers, as opposed to mobile phones, which are often the modality used by patients. Over two-thirds of Americans now own a mobile phone, and 62% of mobile phone owners use their mobile phone to get information about a health care condition [14]. In light of this, it is possible that the search engine may have organized websites differently, and the websites themselves may be navigated differently for mobile phone users. We used a validated questionnaire specifically designed to assess the quality of patient-targeted health information for treatment choices. There are other elements of website design that could meaningfully impact the user experience and patient education that we did not assess. These include ease of readability and use of illustrative graphics. Neither the ECT nor the DISCERN tool include patient perspectives in what they would define as a high-quality website, and we acknowledge that patients may desire different content within websites than physicians. We did not take into account the idea that parents have different values and the role of incorporating their values into decision making regarding periviable delivery, but these are important areas of future research. Finally, we did not consider the Internet self-efficacy or health literacy of our population. These are important areas for future investigation.

Although not the major focus of our study, 90% of our survey population indicated that in addition to high-quality information about prognosis and treatment for periviable neonates, they also wanted to learn about other patients’ experiences with periviable birth and periviable decision making. This suggests that there is a patient desire for the inclusion of narratives in the educational component of preference-sensitive counseling and that providing these narratives in a balanced fashion may be important. It also speaks to potential gaps in counseling by health care providers. Understanding these gaps is an important research effort of our group as well as others [12]. We hope that the information generated by this pilot study will lead to a larger, comprehensive review of online resources available to patients facing a periviable birth.

### Conclusions

Moving forward, the creation of an evidence-based Internet resource that addresses both short- and long-term neonatal outcomes, patients’ values, the importance of shared decision making, and the option for comfort care should be developed to help parents make treatment decisions when facing a periviable delivery.

## Appendix

Multimedia Appendix 1 The 10 essential topics pertinent to periviable birth.

## Abbreviations

ECT: Essential Content Tool
IQR: interquartile range

## References

[1] Younge N, Goldstein RF, Bann CM, Hintz SR, Patel RM, Smith PB, Bell EF, Rysavy MA, Duncan AF, Vohr BR, Das A, Goldberg RN, Higgins RD, Cotten CM, Eunice Kennedy Shriver National Institute of Child Health and Human Development Neonatal Research Network (2017). Survival and neurodevelopmental outcomes among periviable infants. N Engl J Med.

[2] Stoll BJ, Hansen NI, Bell EF, Shankaran S, Laptook AR, Walsh MC, Hale EC, Newman NS, Schibler K, Carlo WA, Kennedy KA, Poindexter BB, Finer NN, Ehrenkranz RA, Duara S, Sánchez PJ, O'Shea TM, Goldberg RN, Van Meurs KP, Faix RG, Phelps DL, Frantz ID, Watterberg KL, Saha S, Das A, Higgins RD, Eunice Kennedy Shriver National Institute of Child Health and Human Development Neonatal Research Network (2010). Neonatal outcomes of extremely preterm infants from the NICHD Neonatal Research Network. Pediatrics.

[3] Hintz S, Kendrick D, Vohr B, Poole W, Higgins R, National Institute of Child Health and Human Development Neonatal Research Network (2005). Changes in neurodevelopmental outcomes at 18 to 22 months' corrected age among infants of less than 25 weeks' gestational age born in 1993-1999. Pediatrics.

[4] Hintz S, Kendrick D, Wilson-Costello D, Das A, Bell EF, Vohr BR, Higgins RD, NICHD Neonatal Research Network (2011). Early-childhood neurodevelopmental outcomes are not improving for infants born at <25 weeks' gestational age. Pediatrics.

[5] Mercier CE, Dunn MS, Ferrelli KR, Howard DB, Soll RF, Vermont Oxford Network ELBW Infant Follow-Up Study Group (2010). Neurodevelopmental outcome of extremely low birth weight infants from the Vermont Oxford network: 1998-2003. Neonatology.

[6] Rysavy M, Li L, Bell E, Das A, Hintz SR, Stoll BJ, Vohr BR, Carlo WA, Shankaran S, Walsh MC, Tyson JE, Cotten CM, Smith PB, Murray JC, Colaizy TT, Brumbaugh JE, Higgins RD, Eunice Kennedy Shriver National Institute of Child Health and Human Development Neonatal Research Network (2015). Between-hospital variation in treatment and outcomes in extremely preterm infants. N Engl J Med.

[7] Kusuda S, Fujimura M, Uchiyama A, Totsu S, Matsunami K, Neonatal Research Network‚ Japan (2012). Trends in morbidity and mortality among very-low-birth-weight infants from 2003 to 2008 in Japan. Pediatr Res.

[8] Stoll B, Hansen N, Bell E, Walsh MC, Carlo WA, Shankaran S, Laptook AR, Sánchez PJ, Van Meurs KP, Wyckoff M, Das A, Hale EC, Ball MB, Newman NS, Schibler K, Poindexter BB, Kennedy KA, Cotten CM, Watterberg KL, D'Angio CT, DeMauro SB, Truog WE, Devaskar U, Higgins RD, Eunice Kennedy Shriver National Institute of Child Health and Human Development Neonatal Research Network (2015). Trends in care practices, morbidity, and mortality of extremely preterm neonates, 1993-2012. JAMA.

[9] Kaempf J, Tomlinson S, Campbell B, Ferguson L, Stewart V (2009). Counseling pregnant women who may deliver extremely premature infants: Medical care guidelines, family choices, and neonatal outcomes. Pediatrics.

[10] Ohlinger J, Kantak A, Lavin J, Fofah O, Hagen E, Suresh G, Halamek LP, Schriefer JA (2006). Evaluation and development of potentially better practices for perinatal and neonatal communication and collaboration. Pediatrics.

[11] Grobman W, Kavanaugh K, Moro T, DeRegnier R, Savage T (2010). Providing advice to parents for women at acutely high risk of periviable delivery. Obstet Gynecol.

[12] Tucker Edmonds B, Savage T, Kimura R, Kilpatrick S, Kuppermann M, Grobman W, Kavanaugh K (2019). Prospective parents' perspectives on antenatal decision making for the anticipated birth of a periviable infant. J Matern Fetal Neonatal Med.

[13] Raju T, Mercer B, Burchfield D, Joseph G (2014). Periviable birth: Executive summary of a joint workshop by the Eunice Kennedy Shriver National Institute of Child Health and Human Development, Society for Maternal-Fetal Medicine, American Academy of Pediatrics, and American College of Obstetricians and Gynecologists. Obstet Gynecol.

[14] Smith A (2015). Pew Research Center.

[15] van Deursen AJ (2012). Internet skill-related problems in accessing online health information. Int J Med Inform.

[16] Charnock D, Shepperd S (2004). Learning to DISCERN online: Applying an appraisal tool to health websites in a workshop setting. Health Educ Res.

[17] Silberg W, Lundberg G, Musacchio R (1997). Assessing, controlling, and assuring the quality of medical information on the Internet: Caveant lector et viewor--Let the reader and viewer beware. JAMA.

[18] Boyer C, Selby M, Appel R (1998). The Health On the Net Code of Conduct for medical and health web sites. Stud Health Technol Inform.

[19] Ghita ML, Loiz D, Petrescu P (2014). Advanced Web Ranking.

[20] Charnock D, Shepperd S, Needham G, Gann R (1999). DISCERN: An instrument for judging the quality of written consumer health information on treatment choices. J Epidemiol Community Health.

[21] Cummings J, Committee on Fetus and Newborn (2015). Antenatal counseling regarding resuscitation and intensive care before 25 weeks of gestation. Pediatrics.

[22] Ecker JL, Kaimal A, Mercer BM, Blackwell SC, deRegnier RA, Farrell RM, Grobman WA, Resnik JL, Sciscione AC, American College of Obstetricians and Gynecologists and the Society for Maternal–Fetal Medicine (2015). #3: Periviable birth. Am J Obstet Gynecol.

[23] South Australian Perinatal Practice Guidelines Workgroup (2013). South Australian Perinatal Practice Guidelines. Parent Information: For Babies Born 23-24 Weeks.

[24] Gandhi A (2014). Patient.

[25] Butler AS, Behrman RE, Institute of Medicine (2007). Preterm Birth: Causes, Consequences, and Prevention.

[26] Yates E, Dixon L (2015). PageRank as a method to rank biomedical literature by importance. Source Code Biol Med.

[27] Bruce J, Tucholka J, Steffens N, Neuman H (2015). Quality of online information to support patient decision-making in breast cancer surgery. J Surg Oncol.

[28] Borgmann H, Wölm JH, Vallo S, Mager R, Huber J, Breyer J, Salem J, Loeb S, Haferkamp A, Tsaur I (2017). Prostate cancer on the Web: Expedient tool for patients' decision-making?. J Cancer Educ.

