# Supplementary figures and images for: Online Information About Periviable Birth: Quality Assessment
Source: JMIR Pediatr Parent. 2019 Jun 7;2(1):e12524. doi: 10.2196/12524 (PMC6716431; doi:10.2196/12524)

## Slide 1
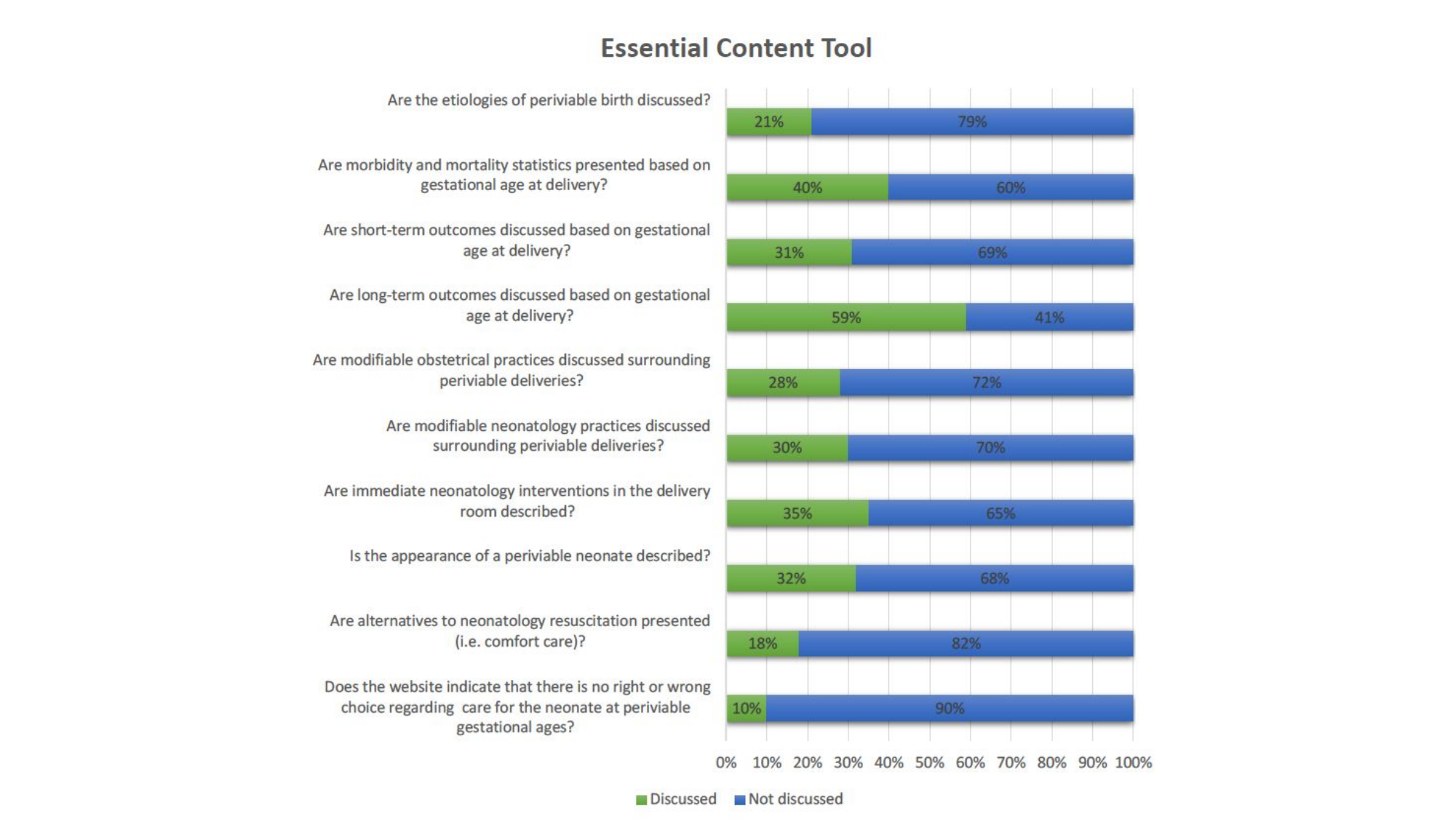

Supplement: Multimedia Appendix 1 [file pediatrics_v2i1e12524_app1.pptx]
